# DNA-Binding Properties of YbaB, a Putative Nucleoid-Associated Protein From *Caulobacter crescentus*

**DOI:** 10.3389/fmicb.2021.733344

**Published:** 2021-10-28

**Authors:** Parul Pal, Malvika Modi, Shashank Ravichandran, Ragothaman M. Yennamalli, Richa Priyadarshini

**Affiliations:** ^1^Department of Life Sciences, School of Natural Sciences, Shiv Nadar University, Gautam Buddha Nagar, India; ^2^Department of Bioinformatics, School of Chemical and Biotechnology, SASTRA Deemed to be University, Thanjavur, India

**Keywords:** DNA binding protein, *Caulobacter crescentus*, nucleoid associated protein, YbaB/EbfC family, gene regulation

## Abstract

Nucleoid-associated proteins (NAPs) or histone-like proteins (HLPs) are DNA-binding proteins present in bacteria that play an important role in nucleoid architecture and gene regulation. NAPs affect bacterial nucleoid organization via DNA bending, bridging, or forming aggregates. EbfC is a nucleoid-associated protein identified first in *Borrelia burgdorferi*, belonging to YbaB/EbfC family of NAPs capable of binding and altering DNA conformation. YbaB, an ortholog of EbfC found in *Escherichia coli* and *Haemophilus influenzae*, also acts as a transcriptional regulator. YbaB has a novel tweezer-like structure and binds DNA as homodimers. The homologs of YbaB are found in almost all bacterial species, suggesting a conserved function, yet the physiological role of YbaB protein in many bacteria is not well understood. In this study, we characterized the YbaB/EbfC family DNA-binding protein in *Caulobacter crescentus. C. crescentus* has one YbaB/EbfC family gene annotated in the genome (YbaB_*C*__*c*_) and it shares 41% sequence identity with YbaB/EbfC family NAPs. Computational modeling revealed tweezer-like structure of YbaB_*C*__*c*_, a characteristic of YbaB/EbfC family of NAPs. N-terminal–CFP tagged YbaB_*C*__*c*_ localized with the nucleoid and is able to compact DNA. Unlike *B. burgdorferi* EbfC protein, YbaB_*C*__*c*_ protein is a non-specific DNA-binding protein in *C. crescentus*. Moreover, YbaB_*C*__*c*_ shields DNA against enzymatic degradation. Collectively, our findings reveal that YbaB_*C*__*c*_ is a small histone-like protein and may play a role in bacterial chromosome structuring and gene regulation in *C. crescentus.*

## Introduction

Similar to eukaryotic organisms, bacteria also pack their genetic material in a very small space. DNA-binding proteins known as nucleoid-associated proteins (NAPs) play a crucial role in nucleoid structuring and controlling gene expression. Although NAPs are referred to as histone-like proteins (HLPs), they are functionally very distinct from eukaryotic histones. Bacterial cells contain a wide variety of NAPs and their expression in the cell varies with growth phase of the culture. NAPs are small, basic proteins that can have the ability to bind to DNA, either as monomers, dimers, or tetramers or in complex with other DNA modulating proteins (Dorman, 2009; Dillon and Dorman, 2010). The DNA binding occurs either through direct physical interaction or indirectly via binding with other accessory proteins (Dorman, 2014).

Major NAPs involved in nucleoid structuring are HU, IHF, H-NS, Lrp, Fis, and Dps. HU is small, basic, non-sequence specific DNA-binding protein, well conserved in eubacteria (Kamashev and Rouviere-Yaniv, 2000; Bahloul et al., 2001). In dimorphic bacterium, *Caulobacter crescentus*, HU is a 20-kDa heterodimer consisting of HU1 and HU2 subunits (Le et al., 2013; Le and Laub, 2016). HU protein colocalizes with the nucleoid and has uniform distribution among swarmer and stalked cells but is highly clustered in predivisional cells (Lee et al., 2011). Recently, a new NAP, GapR, was discovered in *C. crescentus* (Ricci et al., 2016; Arias-Cartin et al., 2017; Taylor et al., 2017). GapR binds AT-rich regions of the nucleoid (Ricci et al., 2016) and absence of GapR leads to morphological and cell division defects (Arias-Cartin et al., 2017; Taylor et al., 2017).

A new family of NAPs, EbfC/YbaB, was first discovered in *Borrelia* (Babb et al., 2006). The structure of EbfC/YbaB homodimer has been described to have a tweezer-like conformation, with tweezer region ascribed to alpha-helical DNA-binding domain and giving spacer region the ability to fit around double-stranded DNA (Lim et al., 2002; Riley et al., 2009). EbfC protein binding facilitates DNA bending (Riley et al., 2009). In *B. burgdorferi*, EbfC displays sequence specific DNA binding and binds to a palindromic DNA sequence, 5′-GTnAC-3′, where “n” can be any nucleotide (Riley et al., 2009). However, the DNA binding sites EbfC/YbaB orthologs of *E. coli* and *H. influenzae* are not known and are believed to be distinct from that of *B. burgdorferi* EbfC (Cooley et al., 2009). While EbfC/YbaB is almost present in all eubacteria, its physiological role in most bacterial species remains largely unknown. Here, we characterized YbaB_*C*__*c*_, the homolog of YbaB/EbfC family DNA-binding protein in *C. crescentus*. YbaB_*C*__*c*_ protein shares 41% sequence identity and has distinctive tweezer-like conformation of ‘YbaB/EbfC family proteins. Moreover, YbaB_*C*__*c*_ colocalizes with nucleoid and is able to compact DNA. YbaB_*C*__*c*_ is a non-sequence specific DNA-binding protein with nucleoid-associated function in *C. crescentus.*

## Materials and Methods

### Strains, Media, and Growth Conditions

*C. crescentus* strains were grown in peptone yeast extract (PYE) medium or M2G minimal medium at 30°C (Stove Poindexter’, 1964) and *E. coli* cells were grown in LB medium at 37°C. Xylose (0.3%), glucose (0.2%), or arabinose (0.2%) was added to the growth media as required. Plasmids and strains used in this study are detailed in Table 1. Details of strain construction are mentioned in Supplementary Text. All media were purchased from Hi-Media Laboratories (Mumbai, India) and antibiotics were obtained from Sigma (United States).

**TABLE 1:** Strains and plasmids.

	Relevant genotype or description	Sources/References
***C. crescentus***		
CB15N	Synchronizable derivative of wild-type strain CB15 (NA 1000)	Evinger and Agabian, 1977
CJW2141	CB15N *cc2570*:pTC67 *ftsI*(Ts)	Costa et al., 2008
RP42	CB15N *xyl*X:pXCFPN-5-*ybab*_*Cc*_	This work
RP43	CB15N/pPAL4	This work
RP45	CB15N *cc2570*:pTC67 *ftsI*(Ts) *xyl*X:pXCFPN-5-*ybab*_*Cc*_	This work
***E. coli***		
DH5α	Φ80 Δ*lacZ*Δ*M15*Δ(lacZYA-argF)U169 *deoR recA1 endA hsdR17 (rk-,mk* +) *phoA supE44 thi-1 gyrA96 relA1*	Laboratory strain collection
S17	RP4-2, Tc:Mu, KM-Tn7	Simon et al., 1983
BL21 (DE3) pLysS	F^–^ *omp*T *hsd*S_*B*_ (r_*B*_^–^, m_*B*_^–^) *gal dcm* (DE3) pLysS(Cam^*R*^)	Laboratory strain collection
MG1655	K-12 F^–^ λ^–^ *ilvG*^–^ *rfb-50 rph-1*	Laboratory strain collection
RP40	MG1655/pPAL1	This work
RP41	BL21 (DE3) pLysS/pPAL3	This work
RP44	BL21 (DE3) pLysS/pPAL5	This work
RP46	MG1655/pBAD18	This work
**Plasmids**		
pBAD18	Kan^*r*^, DH5α containing empty pBAD18	Guzman et al., 1995
pXCFPN-5	Tetr, vector used for generating N-terminal protein fusions encoded at the *xylX* locus	Thanbichler et al., 2007
pET28b	Protein expression vector, Kan^*r*^	Laboratory strain collection
pJS14	High copy number vector, pBR1MCS derivative, Cmr	Laboratory strain collection
pPAL1	pBAD18 carrying *ybab*_*Cc*_, Kan^*r*^	This work
pPAL2	pXCFPN-5 carrying *ybab*_*Cc*_fused to CFP	This work
pPAL3	pET28b carrying *ybab*_*Cc*_fused to 6X-His at N-terminus	This work
pPAL4	pJS14 carrying *ybab*_*Cc*_	This work
pPAL5	pET28b carrying truncated *ybab*_*Cc*_fused to 6X-His at N-terminus	This work

### Microscopy and Image Analysis

Expression from *Pxyl* promoter was obtained by addition of xylose to the growth media at an optical density OD_600_ 0.2. For studying localization of YbaB_*C*__*c*_, expression of CFP-fused YbaB_*C*__*c*_ was obtained from pXCFPN-5 vector in PBP3 temperature-sensitive mutant *C. crescentus* by addition of xylose (0.2%) at an optical density OD_600_ 0.1 in PYE broth. Cultures were incubated at 30°C for 1 h and then shifted to 37°C and grown for 4.5 h. After this, cells were harvested and washed with M2 medium and processed for 4′, 6′-diamidino-2-phenylindole (DAPI) staining. Cell samples (5 μl) were imaged as described before (Dubey and Priyadarshini, 2018) using Nikon Eclipse Ti microscope (United States) equipped with Nikon DS-U3 camera. Images were processed with Adobe Photoshop CS6. Cell length, nucleoid length, and cell width were analyzed with Fiji (ImageJ) software (Schindelin et al., 2012) and Oufti software (Paintdakhi et al., 2015).

### 4′, 6′-diamidino-2-phenylindole Staining

For DNA compaction studies, YbaB_*Cc*_ was ectopically expressed in wild-type *E. coli* MG1655 cells, as described previously with necessary modifications (Ghosh et al., 2013; Oliveira Paiva et al., 2019). *C. crescentus* YbaB_*C*__*c*_ was cloned in pBAD18 and transformed into *E. coli* MG1655. *E. coli* cells containing YbaB_*C*__*c*_ and *E. coli* control strain carrying empty pBAD18 vector were grown overnight at 37°C in LB medium supplemented with 0.2% glucose and kanamycin. Overnight cells were washed twice with LB broth and secondary cultures were induced with 0.2% arabinose or glucose at OD_600_ 0.1. The cells were allowed to grow for 6 h and harvested. Pellets were washed with 1X PBS, fixed in 70% ethanol for 2 min (10% ethanol used in case of colocalization studies with CFP) at room temperature and washed again with 1X PBS. DAPI (1 mg/ml) was added to the cells (1:1,000 dilution) and incubated in dark for 15 min at room temperature. After final wash with 1X PBS, cells were observed by microscopy. For DNA compaction studies, images were captured by Nikon Eclipse Ti 2 Confocal Microscope (AR1MP; United States). Images were processed with Adobe Photoshop CS6. Cell length, nucleoid length, and cell width were analyzed with Fiji (ImageJ) software (Schindelin et al., 2012).

### Protein Purification

The coding regions of YbaB_*C*__*c*_ and YbaB_*C*__*c*_ (Tn) were amplified by PCR and inserted into pET28b(+) (Novagen) between *Sac*I and *Bam*HI sites. These recombinant vectors were used to express 6X His-tagged YbaB_*C*__*c*_ and YbaB_*C*__*c*_ (Tn) proteins in *E. coli* BL21 (DE3) pLysS cells. Recombinant protein expression was obtained by adding 10 μM IPTG to 0.4 OD_600_ bacterial culture for 5 h at 37°C in LB medium supplemented with kanamycin. Cells were harvested by centrifugation (10,000 rpm, 5 min, 4°C) and processed for protein purification as described previously with minor modifications (Dubey and Priyadarshini, 2018). The washed pellets were resuspended in the lysis buffer (50 mM Tris–HCl pH 7.5, 100 mM NaCl, 5% glycerol, 5 mM imidazole, 1 mM PMSF) and incubated with 1 mg/ml lysozyme for 45 min in shaker incubator at 37°C. Cells were lysed by sonication and the lysate was clarified by centrifugation (16,000 rpm, 20 min, 4°C). The supernatant was treated with DNaseI (New England Biolabs, United States) for 15 min at 4°C. Supernatant was incubated with Ni^2+^–NTA agarose beads (Qiagen, Germany) equilibrated in binding buffer (50 mM Tris–HCl pH 7.5, 100 mM NaCl, 5% glycerol and 10 mM imidazole) for 6 h at 4°C. The mixture was passed through a gravity-flow column and washed with wash buffer (50 mM Tris–HCl pH 7.5, 100 mM NaCl, 5% glycerol, and 20 mM imidazole). 6X His-tagged YbaB_*C*__*c*_ was eluted with elution buffer (50 mM Tris–HCl pH 7.5, 100 mM NaCl, 5% glycerol, and 300 mM imidazole) in multiple fractions. Eluted fractions were visualized on SDS–PAGE by Coomassie staining (Supplementary Figure 3). Protein containing fractions were pooled and given a buffer exchange for Storage buffer (50 mM Tris–HCl pH 7.5, 100 mM NaCl, 5% glycerol), using PD-10 Desalting column (Cytiva, United States). Finally, the protein fraction was concentrated using Amicon^®^ Ultra-4 spin-columns (Millipore, United States). Dot blot and Western blot analysis were performed with 1:5,000 dilution anti-His-antibody (Invitrogen, United States; Supplementary Figure 3). Protein concentration was estimated by Bradford Method (Bradford, 1976).

### Western Blot and Dot Blot Analysis

Western blots were performed as described (Dubey and Priyadarshini, 2018). Cell lysates were separated on SDS–PAGE gels and transferred to a polyvinylidene difluoride (PVDF) membrane using a semi-dry transfer apparatus (Bio-Rad, United States). Dot blot was performed as previously described (Bhat and Rao, 2020), with appropriate modifications. Concentrated and diluted purified YbaB_*Cc*_ was spotted onto nitrocellulose membrane and samples were allowed to completely air dry. Membranes were incubated with PBST blocking solution containing 5% non-fat milk at room temperature for 3 h. The membranes were then washed and subsequently incubated with 1:5,000 diluted anti-His antibody (Invitrogen, United States) overnight at 4°C. The next day, membranes were given four 10-min washes in PBST and incubated with horseradish peroxidase-conjugated anti-rabbit antibody (Invitrogen, United States) for 1–2 h at room temperature. Blots were washed with PBST and developed with the Bio-Rad Clarity and Clarity Max ECL Western Blotting Substrates according to the manufacturer’s protocols. 6X His-tagged (Supplementary Figures 3B,C, 4) fusion proteins were confirmed by western blotting for stability.

### DNA Protection Assay

To investigate DNA binding and protection ability of YbaB_*C*__*c*_ against degradative action of DNases, pBAD18-kan vector was incubated with increasing YbaB_*C*__*c*_ protein concentrations (20 min, 25°C) in Storage Buffer. The experiments were performed as described earlier (Datta et al., 2019). In brief, enzyme treatment was given at 37°C for 1 min using 1 Unit of DNaseI (New England Biolabs, United States). The enzyme was inactivated by incubation at 75°C for 15 min followed by protein denaturation at 95°C for 20 min. Reactions were electrophoresed in 1% agarose gel containing ethidium bromide stain and DNA was visualized in a UV transilluminator.

### Electrophoretic Mobility Shift Assay

EMSA was performed according to LightShift Chemiluminescent EMSA Kit Protocols, (Thermo Scientific, United States). In brief, oligonucleotides b-WT (124 bp) from *Borrelia burgdorferi* strain B31 (Riley et al., 2009) and cc-WT (200 bp of *ybab_*C*__*c*_* gene) from *C. crescentus* (this work) were labeled at 3′ end with Biotin-11-UTP according to the manufacturer’s protocol (Biotin 3′ end DNA Labeling Kit, Thermo Scientific, United States). Labeled target probes were incubated with increasing concentrations of purified YbaB_*C*__*c*_ in a reaction containing binding buffer (LightShift Chemiluminescent EMSA Kit, Thermo Scientific) and 50% glycerol at 25°C for 30 min. The reactions were electrophoresed in 6% DNA retardation gel (Invitrogen, United States) in 0.5x TBE. Separated DNA products were then electroblotted onto a positively charged Nylon membrane (Thermo Fisher Scientific, United States) using a semi-dry transfer apparatus (Bio-Rad, United States). Cross-linking by UV was done at 120 mJ/cm^2^ for 1 min immediately after electroblotting. Detection of DNA and DNA-protein complexes was carried using Chemiluminescent Nucleic Acid Detection Module (Thermo Scientific, United States).

### Building Computational Model of YbaB_*Cc*_ and Its Complex With DNA

The protein sequence of YbaB_*Cc*_ was retrieved from NCBI (accession id: YP_002515644.1) and was given as input to Robetta tool (Kim et al., 2004),^1^ where the comparative modeling approach was used to generate 1,000 sample models and obtained the top five models (Kim et al., 2004). The five models were analyzed to select one model using root mean square deviation as a selection measure. Additionally, the model’s confidence score was also taken into consideration for building the protein-DNA complex. The DNA sequence (5′-ATGTAACAGCTGAATGTAACAA-3′) was used to construct a double-stranded B-DNA and the 3D coordinates were obtained using the conformational parameters extracted from fiber-diffraction experimental studies.^2^ Subsequently, the protein and DNA were given as input to HADDOCK (van Zundert et al., 2016).

### Protein-DNA Docking Studies

The parameters of protein-DNA docking were selected in the manner to perform a blind docking approach, where specific interaction site was not specified (Heté Nyi and van der Spoel, 2002; Teotia et al., 2019; Gondil et al., 2020). Additional parameters were set, such as force constant for center of mass contact restraints (1.0), force constant for surface contact restraints (1.0), radius of gyration (17.78), number of structures of rigid body docking (1,000), number of trials of rigid body minimization (5), sample 180 rotated solutions during rigid body EM (Yes), number of structures for semi-flexible refinement (200), sample 180 rotated solutions during semi-flexible SA (No), Perform final refinement (Yes), number of structures for the final refinement (200), number of structures to analyze (200), Fraction of Common Contacts (FCC) method of clustering method, RMSD cutoff for clustering (0.6), minimum cluster size (4), Non-bonded parameters (OPLX), include electrostatic during rigid body docking (Yes), Cutoff distance to define an hydrogen bind (2.5), cutoff distance to define a hydrophobic contact (3.9), Perform cross-docking (Yes), randomize starting orientations (Yes), perform initial rigid body minimization (Yes), allow translation in rigid body transformation (Yes). The top ranked protein-DNA complex from HADDOCK was analyzed using NUCPLOT (Luscombe et al., 1997).

## Results

### Protein–DNA Complex Indicates Preferential Binding of YbaB_*C*__*c*_

**FIGURE 1:**
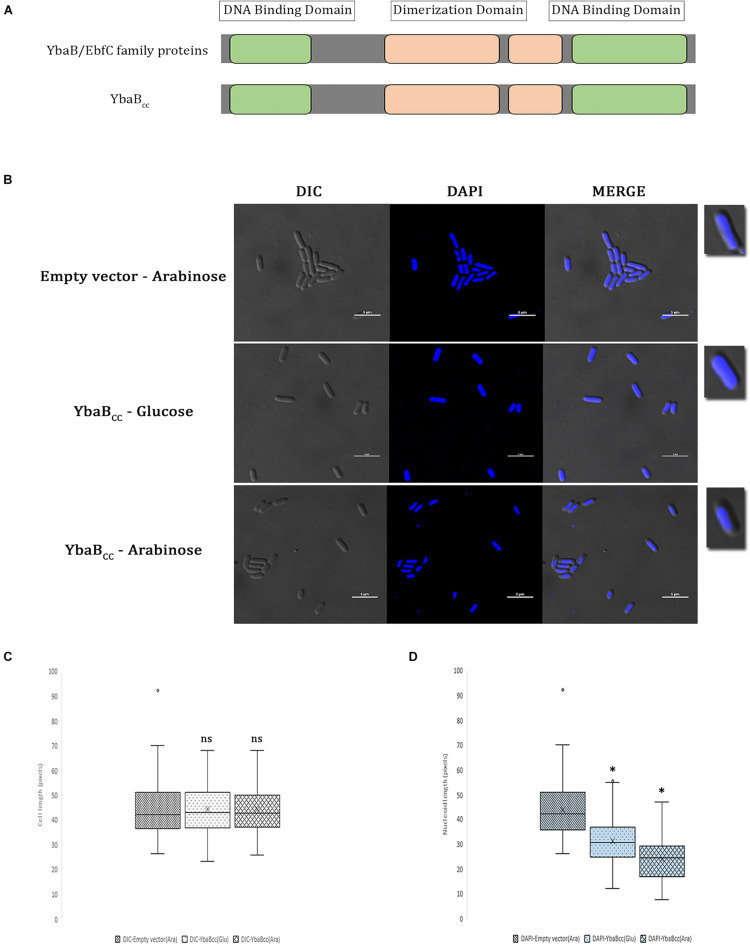
YbaB_*Cc*_ binds and compacts DNA in *E. coli*. **(A)** Schematic presentation of YbaB_*Cc*_ protein domain organization. YbaB/EbfC family proteins show similarity in domain structures with YbaB_*Cc*_. There are two DNA-binding domains present at either terminus of YbaB/EbfC family proteins, which together form a unique tweezer-like structure in order to bind to the target DNA. **(B)** YbaB_*Cc*_ overexpression in *E. coli* cells. YbaB_*Cc*_ was expressed ectopically from pBAD promoter in RP40 cells. In wild-type *E. coli*, nucleoid occupies the entire cytoplasmic space, whereas a compacted nucleoid tends to be away from the cell periphery and more toward the center of the bacterial cell. The scale bar represents 5 μM. **(C)** Box and whisker plots of mean cell length. Whiskers represent minimum and maximum cell lengths observed in each strain (1 μM = 32 pixels). Cross (x) represents mean values and horizontal line across boxes represent the median values. Analysis was done using Fiji (ImageJ) software (n, number of cells analyzed = 99 per strain, per condition). **(D)** Box and whisker plots of mean nucleoid length. Whiskers represent minimum and maximum nucleoid lengths observed in each strain (1 μM = 32 pixels). Cross (x) represents mean values and horizontal line across boxes represent the median values. Analysis was done using Fiji (ImageJ) software (n, number of cells analyzed = 99 per strain, per condition). **(C,D)** Same cells were analyzed for measuring cell lengths and nucleoid lengths. YbaB_*Cc*_ protein bound nucleoid reduces in length as compared to the protein-free nucleoid in non-overexpressing conditions. Box and whisker plots (*n* = 99 per strain) were generated for statistical analysis with the whiskers including all data points within 1.5^∗^IQR. ns = non-significant. ^∗^*p* < 0.0001 by one-way ANOVA compared to the empty vector pBAD (ara) control.

EbfC/YbaB homologs are ubiquitous in nearly all eubacterial genomes. *C. crescentus* genome CCNA_00269 is annotated as DNA-binding protein and BLAST analysis revealed 41% sequence identity and 62% similarity with EbfC/YbaB family NAPs. EbfC/YbaB family of proteins act as NAPs and have dimerization domains flanked on either side by DNA-binding domains (Babb et al., 2006; Riley et al., 2009). CCNA_00269 revealed similar domain arrangement (Figure 1A). Based on these results we annotated CCNA_00269 gene as *ybab_*C*__*c*_* and characterized its DNA binding properties in this study. EbfC/YbaB family of NAPs colocalize with the nucleoid and probably play a role in structuring of the bacterial chromosome (Jutras et al., 2012; Wang et al., 2012). To investigate DNA compaction activity of YbaB_*C*__*c*_ protein, we introduced full-length YbaB_*C*__*c*_ on pBAD18 plasmid into *E. coli.* Ectopic expression of YbaB_*C*__*c*_ was induced by addition of arabinose and cells were subsequently stained with DAPI to label the nucleoid (Figure 1B). As evident from Figures 1B–D, only 51.3% of cell length is occupied by the nucleoid in arabinose-induced cells. In comparison, *E. coli* cells grown with glucose showed 68.4% of the cell length being occupied by nucleoid. Nucleoid compaction observed in presence of glucose could be due to leaky expression from high copy number pBAD18 plasmid (Dubey and Priyadarshini, 2018). In comparison, control RP46 cells carrying empty pBAD plasmid grown in presence of arabinose displayed almost 99% of the cell length being occupied by nucleoid. Our results suggest that YbaB_*C*__*c*_ protein is able to compact DNA.

**FIGURE 2:**
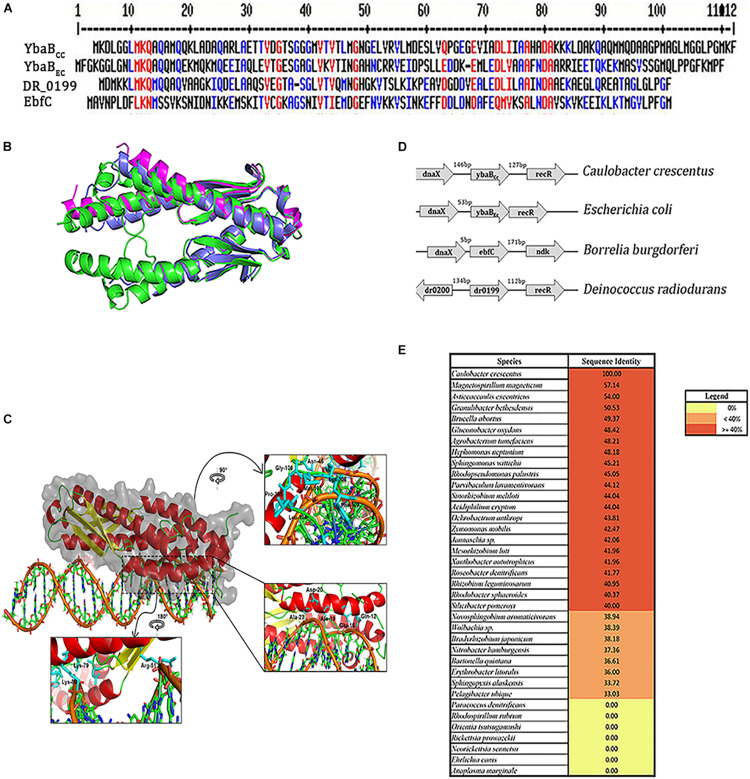
YbaB_*cc*_ is conserved across multiple bacterial species. **(A)** Multiple sequence alignment of YbaB/EbfC proteins from different bacterial species using MultAlin software (red—high consensus residues, blue—low consensus residues, at the given position). **(B)** Structural superposition of the modeled YbaB_*Cc*_ protein with structural homologs. The modeled protein (colored green and shown in cartoon representation) is a homodimer, as seen in the structural homologs from *E. coli* (PDB id: 1PUG) colored light blue. Another structural homolog from *Haemophilus influenza* (PDB id:1J8B) colored magenta is in monomeric form. Image made using PyMOL. **(C)** Protein-DNA docking model of YbaB_*Cc*_ (shown in cartoon and surface representation colored in secondary structure) is observed to bind to the DNA motif (5′-ATGTAACAGCTGAATGTAACAA-3′) as obtained from HADDOCK web server. The insets show the orientation of side chains of the interacting residues in stick representation (colored cyan). **(D)** Schematic representation of the *ybaB*/*ebfC* gene locus with adjacent genes in various bacteria. This arrangement of genes appears to be conserved in most bacteria that harbor YbaB/EbfC proteins. **(E)** YbaB homologs in alpha proteobacteria. Genomes displaying YbaB homologs with more than 40% sequence identity are labeled in red and proteins having identity below 40% are labeled in orange. Bacterial genomes with no YbaB homologs are labeled in yellow.

We further searched for YbaB homologs in other alpha-proteobacteria. YbaB homologs were found in most alpha-proteobacteria except, intracellular pathogens belonging to the family Rickettsiaceae (Figure 2E). *Rhodospirillum rubrum* and *Paracoccus denitrificans* genome were also devoid of YbaB homologs (Figure 2E). EbfC/YbaB family proteins function as homodimers having a unique “tweezer”-like structure (Lim et al., 2002; Cooley et al., 2009). We obtained a 3D model of YbaB_*Cc*_ protein using Robetta program (Kim et al., 2004). Robetta samples nearly 1,000 models obtained after superposing the partial threads and gives the user five highly ranked models for a given sequence (Kim et al., 2004). For YbaB_*Cc*_ protein sequence, we obtained five models that had a confidence score of 0.75. The confidence score is a qualitative indicator for the user to gauge the success of the modeling, where a confidence score of 0.7 and above for a model is considered as high-quality models that are akin to those obtained by low-resolution X-ray crystallography or NMR experiments (Song et al., 2013). Thus, the confidence score of 0.75 for YbaB_*Cc*_-modeled structure indicates a high-quality model and Model 1 was taken as the candidate structure for all further experimental analysis. The 3D structure of YbaB_*Cc*_ was strikingly similar to crystalized YbaB/EbfC structures (Lim et al., 2002) showing characteristic tweezer-like conformation (Figure 2B). DNA-binding properties of Model 1 were probed further. The coordinates of Model 1 and DNA were given as input to HADDOCK and the resulting four top-ranked conformations showed that the protein has a preferential site of binding with the nucleic acid (Figure 2C). To explore further protein-DNA interactions were mapped using NUCPLOT, where for each conformation made both hydrogen and hydrophobic interactions were probed. YbaB_*C*__*c*_ protein models displayed both hydrogen bonds and hydrophobic interactions with DNA (Figure 2C). Further, mapping the electrostatic surface of the modeled YbaB_*Cc*_ protein showed a patch of positively charged surface (sequence range from Arg51 to Ile71, Supplementary Figure 2) that is most likely to interact with DNA. On mapping the electrostatic surface of the modeled YbaB_*C*__*c*_ protein, a patch of positively charged region (sequence range from Arg51 to Ile71, Supplementary Figure 1) was found giving an idea about where the probable binding site is present. Further, the top scoring protein-DNA complex, obtained from HADDOCK web server, was given as input to the NUCPLOT program. Cut-offs of 3 and 3.35 Å were used for hydrogen bond and other non-bonded interactions, respectively. The interaction network constructed predicted 24 residues interact with the DNA forming both hydrophobic as well as hydrogen bonds. The network predicted a single hydrogen bond formed between Lys79 residue and the 31st nucleotide whereas other residues formed other kinds of non-bonded interactions. Multiple sequence alignment (MSA) of YbaB_*Cc*_ was carried out using MultAlin software (Corpet, 1988), which generated the consensus residues (red color) also present in other YbaB/EbfC family proteins from *Borrelia burgdorferi* (EbfC), *E. coli* (YbaB_*Ec*_), and *Deinococcus radiodurans* (DR_0199; Figure 2A). Gln-12 and Lys-80 residues are found to be crucial in DNA docking (Figure 2C) and are also common to all four YbaB/EbfC family proteins assessed by MSA (Figure 2A).

### Localization of YbaB_*C*__*c*_ Protein

**FIGURE 3:**
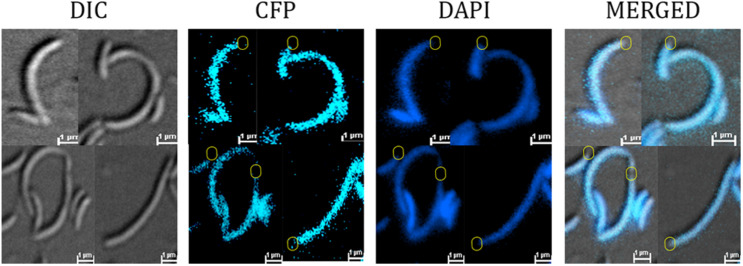
YbaB_*Cc*_ colocalizes with nucleoid *in vivo*. RP44 cells (CB15N *cc2570*:pTC67 *ftsI*(Ts) *xyl*X:pXCFPN-5-*ybab*_*Cc*_) were cultured at 30°C for 1 h and then shifted to the restrictive temperature (37°C) for 4.5 h in PYE medium, followed by DAPI staining and imaging on agarose-padded glass slides. Fluorescence imaging shows YbaB_*Cc*_-CFP colocalizing with DAPI-stained nucleoid in a temperature-sensitive PBP3 mutant *Caulobacter crescentus*. DNA-free regions devoid of both DAPI and CFP signal are depicted in circles (yellow).

To determine the cellular localization of the YbaB_*C*__*c*_ in *Caulobacter* cells, YbaB_*C*__*c*_ was fused with CFP at the N-terminal and expressed from *Pxyl* promoter. RP42 cells showed CFP signal throughout the cell (Supplementary Figure 1). In *C. crescentus* cells the chromosome spreads from pole to pole making it difficult to differentiate between cytoplasmic and NAPs colocalization with DNA (Arias-Cartin et al., 2017). To probe whether YbaB_*C*__*c*_ protein colocalized with the nucleoid, the CFP-YbaB_*C*__*c*_ fusion protein was expressed in a temperature sensitive *ftsI C. crescentus* mutant, which forms filaments at the restrictive temperature. In this mutant, YbaB_*C*__*c*_ colocalized with the DAPI signal and was absent in few DNA-free regions (Figure 3), indicating that YbaB_*C*__*c*_ is a nucleoid associated protein.

### YbaB_*C*__*c*_ Interaction With DNA Is Not Sequence-Dependent

**FIGURE 4:**
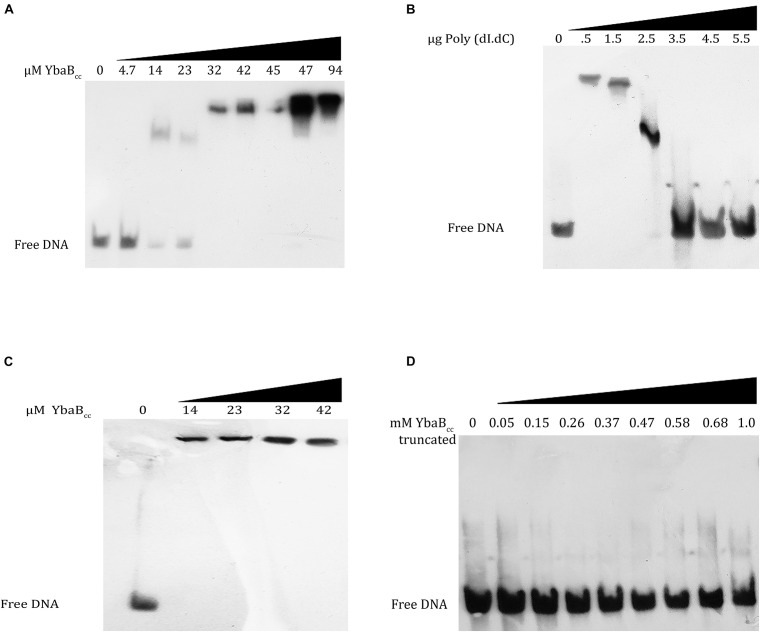
YbaB_*Cc*_ binds to DNA in a sequence-independent manner *in vitro*. YbaB_*Cc*_ was incubated with 8 nM each of biotin-labeled probes. **(A)** b-WT (124 bp) from *Borrelia burgdorferi* strain B31 was incubated with increasing concentrations (0, 4.7, 14, 23, 32, 42, 45, 47, and 94 μM) of YbaB_*Cc*_ protein. **(C)** cc-WT (200 bp) *Caulobacter* probe was incubated with increasing concentrations (0, 14, 23, 32, and 42 μM) of YbaB_*Cc*_ protein. Reactions were electrophoresed on 6% DNA retardation gels and visualized by chemiluminescent detection. YbaB_*Cc*_ forms DNA–protein complexes with both probes **(A–C)** causing shifts of protein–DNA complexes. **(B)** Competitive binding was also studied by adding varied concentrations (0, 0.5, 1.5, 2.5, 3.5, 4.5, and 5.5 μg) of Poly (dI.dC) to YbaB_*Cc*_ (32 μM). The presence of free DNA with increasing Poly (dI.dC) indicates sequence independent DNA-binding activity of YbaB_*Cc*_ by exchange of b-WT (124 bp) from *Borrelia burgdorferi* strainB31 probe with Poly (dI.dC). **(D)** YbaB_*Cc*_ (Tn) protein (containing only N-terminal DNA-binding domain) was also tested for its ability to bind b-WT *Borrelia* probe. However, there was no shift observed even at very high protein concentrations rendering this truncated version of YbaB_*Cc*_ to be ineffective in DNA-binding suggesting that both DNA-binding domains are essential for YbaB_*Cc*_ protein to bind target DNA.

In order to probe the DNA-binding activity of *C. crescentus* YbaB, we purified YbaB_*C*__*c*_ protein, tagged with 6X His at the N-terminal. DNA binding of YbaB_*C*__*c*_ was first tested using biotin labeled DNA probe corresponding to operator sequences of *erpAB* in *B. burgdorferi* (b-WT; Riley et al., 2009). This DNA sequence was selected as EbfC protein from *B. burgdorferi* preferentially binds to sequences within this region. Oligonucleotide b-WT (124 bp) from *Borrelia burgdorferi* strain B31 (Riley et al., 2009) was incubated with increasing concentrations (0, 4.7, 14, 23, 32, 42, 45, 47, and 94 μM) of YbaB_*C*__*c*_ protein for electrophoretic mobility shift assay. YbaB_*C*__*c*_ protein bound to the duplex DNA probes and formed stable protein-DNA complexes (Figure 4A). EbfC preferentially binds to GTnAC sequence found in *erpAB* operator 2 region (Riley et al., 2009). To investigate the specificity of YbaB_*C*__*c*_ binding, we performed EMSA with increasing concentrations of poly (dI-dC) probe as a competitor for non-specific DNA-binding activities. As evident from Figure 4B, concentrations above 2.5 μg of poly (dI-dC) probes (∼60-fold higher or above) were able to abolish YbaB_*C*__*c*_-DNA complexes, indicating the YbaB_*C*__*c*_ in *C. crescentus* might be a non-specific DNA-binding protein. To confirm non-specific DNA-binding activity of YbaB_*C*__*c*_, a 200-bp DNA sequence (cc-WT) of *ybab_*C*__*c*_* gene from *C. crescentus* was amplified and this biotin-labeled DNA duplex was used as probe in EMSA. Even very low concentrations (14 μM) of YbaB_*C*__*c*_ protein caused super shift of cc-WT sequence probe (Figure 4C). Taken together, our data suggests that YbaB_*C*__*c*_ interaction with DNA is not sequence dependent.

*B. burgdorferi* EbfC variants carrying mutations in α-helix 1 or 3 are defective in DNA binding (Riley et al., 2009). *C. crescentus* YbaB_*C*__*c*_ protein, DNA-binding domains are present at both the N- and C-terminus. To investigate if both DNA-binding domains are essential for YbaB_*C*__*c*_ DNA-binding activities, we created a truncated version—YbaB_*C*__*c*_ (Tn), deleted for C-terminal DNA-binding domain and consisting of 1–200 bp of *ybab_*C*__*c*_* gene. YbaB_*C*__*c*_ (Tn) protein was purified and EMSA was performed using b-WT *Borrelia* oligonucleotide. YbaB_*C*__*c*_ (Tn) protein was unable to form stable protein-DNA complexes and no shift was observed (Figure 4D). These results suggest that C-terminal DNA-binding domain of YbaB_*C*__*c*_ is important for DNA-binding activity.

### YbaB_*C*__*c*_ Protects DNA From Degradation

**FIGURE 5:**
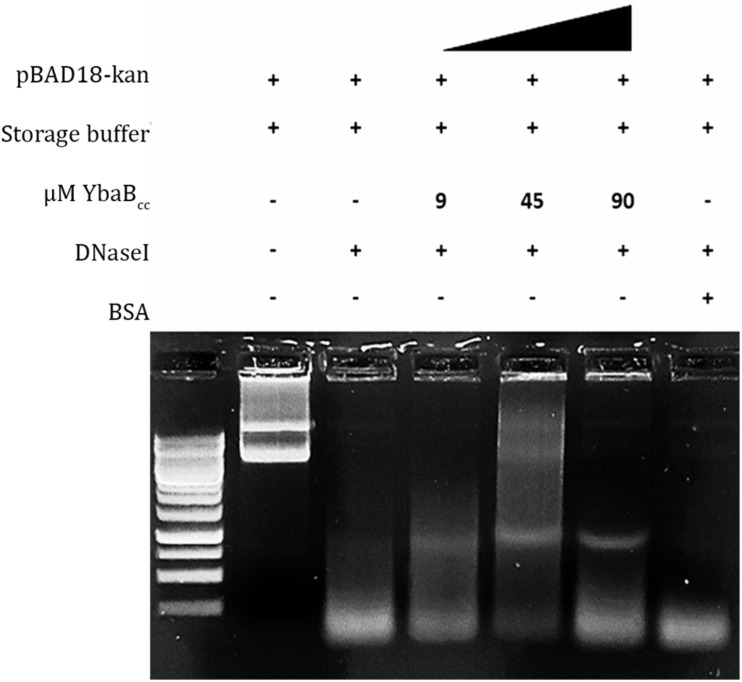
YbaB_*Cc*_ protects DNA from enzyme degradation. Different concentrations of YbaB_*Cc*_ protein were incubated with supercoiled pBAD18 plasmid DNA, followed by treatment with DNase I restriction enzyme. Control reactions were incubated with BSA in place of YbaB_*Cc*_ protein. YbaB_*Cc*_ prevents complete plasmid DNA degradation by the enzyme as opposed to the BSA control reaction, where DNase I is able to degrade the plasmid completely. Thus indicating that YbaB_*Cc*_ protein-bound plasmid DNA was protected by the degradative action of DNase I restriction enzyme and BSA could not protect the plasmid DNA from being degraded as it does not have DNA-binding activity.

Most NAPs in bacteria are able to protect DNA from degradation. We investigated YbaB_*C*__*c*_ DNA protection activity by performing DNase I enzymatic degradation assay. Supercoiled pBAD18 plasmid was incubated with increasing YbaB_*C*__*c*_ protein concentrations and then treated with DNaseI enzyme (Figure 5). As seen in Figure 5, supercoiled plasmid DNA was protected from enzymatic degradation in presence of YbaB_*C*__*c*_. In contrast, the control sample incubated with BSA was degraded upon treatment with DNaseI (Figure 5). Our results indicate that YbaB_*C*__*c*_ protein may protect DNA against damage and degradation.

### Overexpression of YbaB_*C*__*c*_ in Nutrient Limiting Conditions Leads to Morphological Aberrations

**FIGURE 6:**
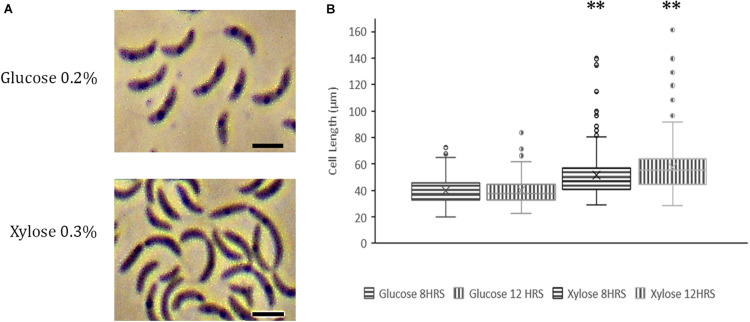
YbaB_*Cc*_ overexpression in wild-type *C. crescentus*. **(A)** Overexpression of YbaB_*cc*_ leads to morphological defects in *C. crescentus*. Expression of YbAB_*Cc*_ from plasmid pJS14 was induced by the addition of 0.3% xylose at an OD of 0.2 and cells were visualized at the indicated time points by Phase Contrast Microscopy (left panel). All images were taken at 100X magnification and optical zoom 1.5X (Scale bar: 2 μm). **(B)** Distribution of cell length in populations of YbaB_*Cc*_ overexpressing cells. Micrographs of the strains described in **(A)** were subjected to automated image analysis. Cell lengths were determined by Oufti Software. Box and whisker plots (*n* = ± 210 per time point and strain) were generated for statistical analysis of imaging data with the whiskers including all data points within 1.5^∗^IQR. Cross (x) represents mean values. ^∗∗^*p* < 0.0001 by one-way ANOVA compared to the glucose-induced control cells.

Abundance of NAPs is highly regulated and fluctuations in protein levels cause adverse effects on cells (Ali Azam et al., 1999). Constitutive overexpression of GapR at high levels is lethal for *C. crescentus* (Ricci et al., 2016). To investigate whether YbaB_*C*__*c*_ overexpression leads to abnormalities, we expressed YbaB_*C*__*c*_ from pJS14 plasmid under the control of xylose promoter. Over expression of YbaB_*C*__*c*_ caused morphological defects in *Caulobacter* cells grown in nutrient limiting conditions. Specifically, we observed filamentous cells and conjoined daughter cells with cell separation defects (Figure 6A). Cell length was increased by 44% compared to control cell grown in glucose (Figures 6A,B). It should be noted that overexpression of YbaB_*C*__*c*_ in cells growing in nutrient rich PYE medium displayed no growth and morphological defects (data not shown). Collectively, our data suggests that YbaB_*C*__*c*_ concentration in regulated in *C. crescentus* and probably plays a critical role in stress response.

## Discussion

Bacterial chromosomes are condensed and stabilized by the action of NAPs. NAPs are also involved in global gene expression by making alterations in DNA structure or by interactions with transcriptional machinery. In this study, we have characterized the DNA-binding properties of YbaB_*C*__*c*_ protein in *C. crescentus*. YbaB_*C*__*c*_ belongs to EbfC/YbaB class of DNA-binding proteins, first discovered in *B. burgdorferi* (Babb et al., 2006). EbfC homodimers have unique tweezer-like structure, where extending α-helices form the arms of the “tweezer” that bind DNA (Lim et al., 2002; Riley et al., 2009). *B. burgdorferi* EbfC protein is a sequence specific NAP and acts as a global regulator of gene expression. However, YbaB_*Ec*_ and YbaB_*H*__*i*_ do not preferentially bind *B. burgdorferi* palindromic DNA sequence (Cooley et al., 2009) EbfC homolog from *D. radiodurans* also acts as a non-specific DNA-binding protein (Wang et al., 2012). Our results indicate that YbaB_*C*__*c*_ might act as a non-specific DNA-binding protein. Differences in DNA binding are attributed to amino-acid differences in the putative DNA-binding domain (Cooley et al., 2009). *C. crescentus* YbaB_*C*__*c*_ exhibits characteristic tweezer-like conformation of EbfC/YbaB family of NAPs and probably functions as a homodimer. Both N and C-terminals of YbaB_*C*__*c*_ protein have DNA-binding domains and deletion of the C-terminus DNA-binding domain is sufficient to abolish DNA-duplex binding activity (Figure 4D).

Similar to *B. burgdorferi* and *D. radiodurans*, YbaB_*C*__*c*_ is associated with the nucleoid in *C. crescentus* (Jutras et al., 2012; Wang et al., 2012; Figure 3). Ectopic expression of YbaB_*C*__*c*_ in *E. coli* condensed the nucleoid suggesting involvement of YbaB_*C*__*c*_ in nucleoid organization and DNA structuring in cells. Collectively our data indicates that YbaB_*C*__*c*_ is a homolog of EbfC/YbaB and has histone-like activity in *C. crescentus*.

NAPs are involved in global gene expression by making alterations in DNA structure or by interactions with transcriptional machinery. HU is one of the most abundant NAP in bacteria involved in nucleoid structuring (Shindo et al., 1992; Ghosh and Grove, 2004; Nguyen et al., 2009; Oberto et al., 2009; Berger et al., 2010). Surprisingly, *C. crescentus* hu deletion mutants deletion mutants display no adverse effect on cell growth, fitness and chromosome architecture (Christen et al., 2011; Lee et al., 2011). Moreover, loss of other NAPs such as IHF and DPS has no fitness cost on *C. crescentus* indicating functional redundancy among NAPs (Christen et al., 2011). Thus, it is not surprising that YbaB_*C*__*c*_ is not essential for growth and viability of *C. crescentus* under standard laboratory conditions (Christen et al., 2011). While *ebfC* is an essential gene in *B. burgdorferi* (Riley et al., 2009; Jutras et al., 2012), deletion of *efbC* homolog from *D. radiodurans* does not affect cell viability (Wang et al., 2012). However, *D. radiodurans dr0199* mutants have increased sensitivity to UV radiation and oxidative stress (Wang et al., 2012).

Role of NAPs in DNA protection is well established. HU from *Thermotoga maritima* and *Helicobactor pylori* shield DNA from endolytic cleavage by DNase I and hydroxyl radical-mediated damage (Mukherjee et al., 2008; Almarza et al., 2015). Lsr2 from *Mycobacterium tuberculosis* acts against reactive oxygen intermediates by directly binding to DNA and shielding the DNA from damage (Colangeli et al., 2009). EbfC homolog from *D. radiodurans* protects double-stranded DNA from digestion and damage from reactive oxygen species (Wang et al., 2012). Similarly, our results reveal that YbaB_*C*__*c*_ protects double-stranded DNA from enzymatic degradation, probably by direct protein-DNA binding (Figure 5). YbaB_*C*__*c*_ may play a crucial role in maintaining genomic integrity by restricting DNA damage in *C. crescentus*.

Most sequenced eubacterial genomes contain adjacent *dnaX* and *ebfC* genes (Flower and Mchenry, 1990; Flower and McHenry, 1991; Chen et al., 1993). In *B. burgdorferi* and *E. coli*, *dnaX* and *ebfC* are arranged in an operon and are co-transcribed together (Riley et al., 2009). *C. crescentus* genome also has *dnaX* gene adjacent to YbaB_*C*__*c*_ (Figure 2D). The *dnaX* gene encodes the tau and gamma subunits of DNA polymerase and YbaB is a NAP with ability to compact and protect DNA (Flower and McHenry, 1991; Chen et al., 1993). Transcriptional linkage of *dnaX* and *ybaB/ebfC* may allow bacterial cells to respond to DNA damage in a timely and regulated manner. Our preliminary experiments to detect transcriptional linkage between *dnaX* and YbaB_*C*__*c*_ did not yield positive results (data not shown). This could be due to low levels of transcripts, as the expression of these genes is growth phase-dependent, or *dnaX* and YbaB_*C*__*c*_ maybe transcribed independently in *C. crescentus*. EbfC levels are highest in exponential phase and rapidly decline in stationary phase in *B. burgdorferi* (Jutras et al., 2012). RecR protein plays crucial role in DNA recombination and repair is also located on the same locus (Figure 2D; Mahdi and Lloyd, 1989; Yeung’ et al., 1990). Similar to YbaB_*C*__*c*_, RecR too is expendable for growth under normal laboratory conditions in *C. crescentus* (Christen et al., 2011). It is interesting to speculate that both YbaB and RecR may play a role in DNA repair and protection during stress conditions. YbaB homologs are present in most alpha proteobacteria, suggesting an important physiological role of this NAP. Further studies are necessary to elucidate the physiological role of YbaB_*C*__*c*_ in *C. crescentus*.

## Data Availability Statement

The original contributions presented in the study are included in the article/Supplementary Material, further inquiries can be directed to the corresponding author/s.

## Author Contributions

RP and PP designed and conceptualized the study. PP and MM performed the experiments. SR and RY performed the bioinformatics analysis. All authors contributed toward the writing of the manuscript.

## Conflict of Interest

The authors declare that the research was conducted in the absence of any commercial or financial relationships that could be construed as a potential conflict of interest.

## Publisher’s Note

All claims expressed in this article are solely those of the authors and do not necessarily represent those of their affiliated organizations, or those of the publisher, the editors and the reviewers. Any product that may be evaluated in this article, or claim that may be made by its manufacturer, is not guaranteed or endorsed by the publisher.

## Footnotes

1
http://new.robetta.org/

2
http://www.scfbio-iitd.res.in/software/drugdesign/bdna.jsp
