# Supplementary figures and images for: DNA-Binding Properties of YbaB, a Putative Nucleoid-Associated Protein From Caulobacter crescentus
Source: Front Microbiol. 2021 Oct 28;12:733344. doi: 10.3389/fmicb.2021.733344 (PMC8581549; doi:10.3389/fmicb.2021.733344)

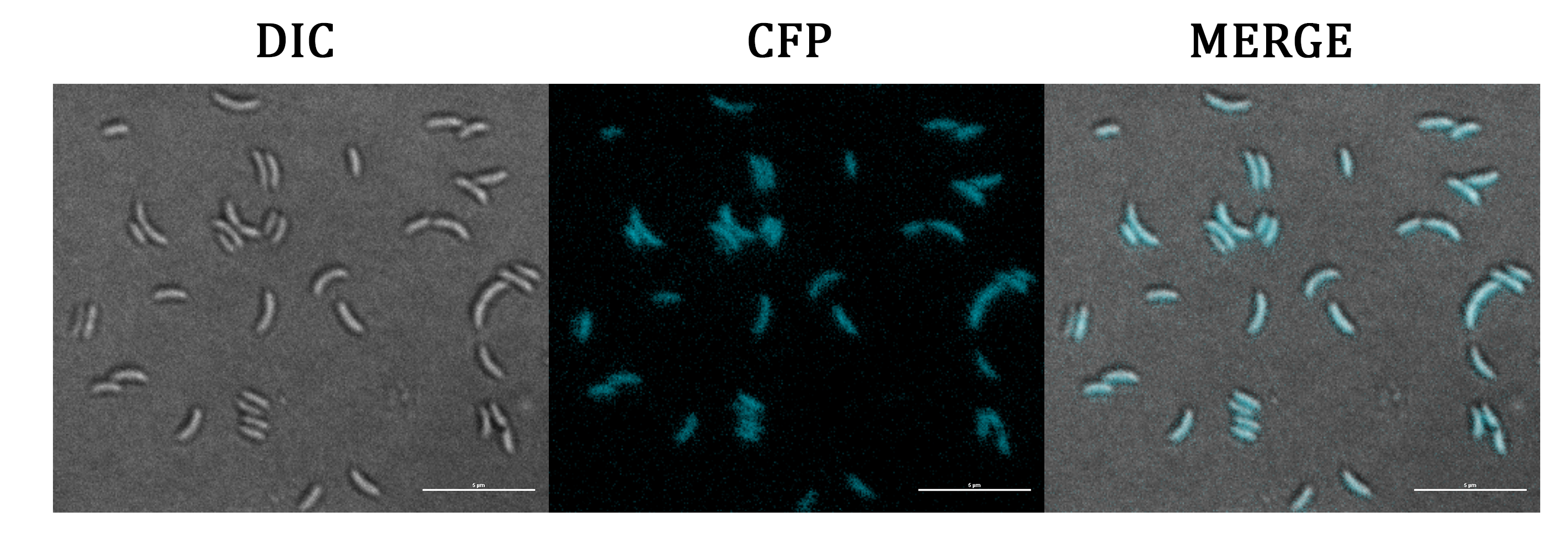

Supplement: Supplementary file 1 [file Image_1.TIF]

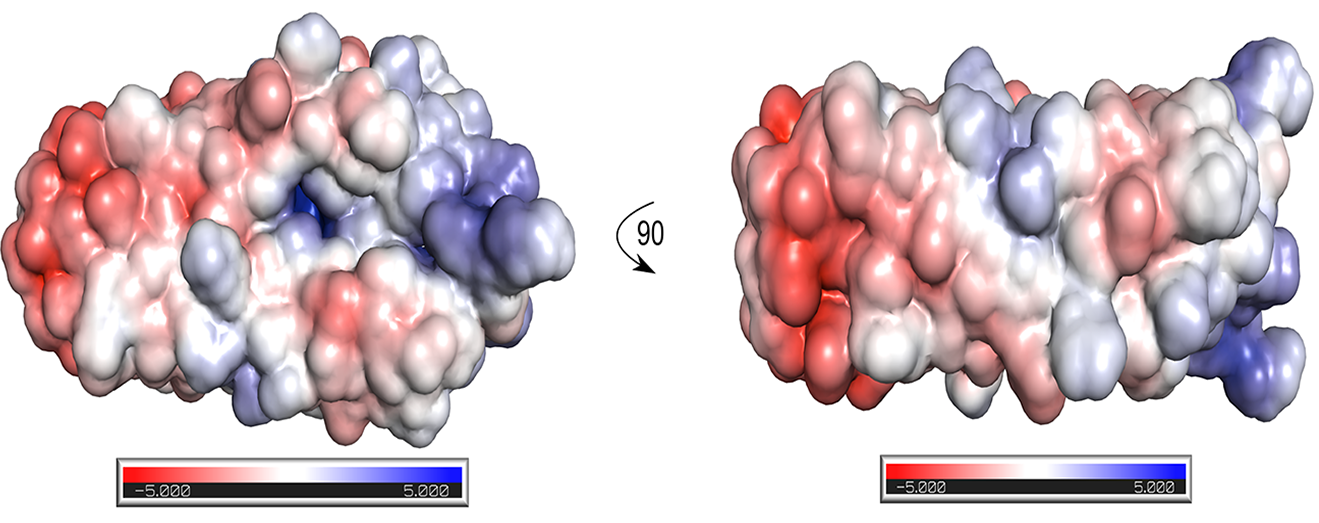

Supplement: Supplementary file 2 [file Image_2.TIF]

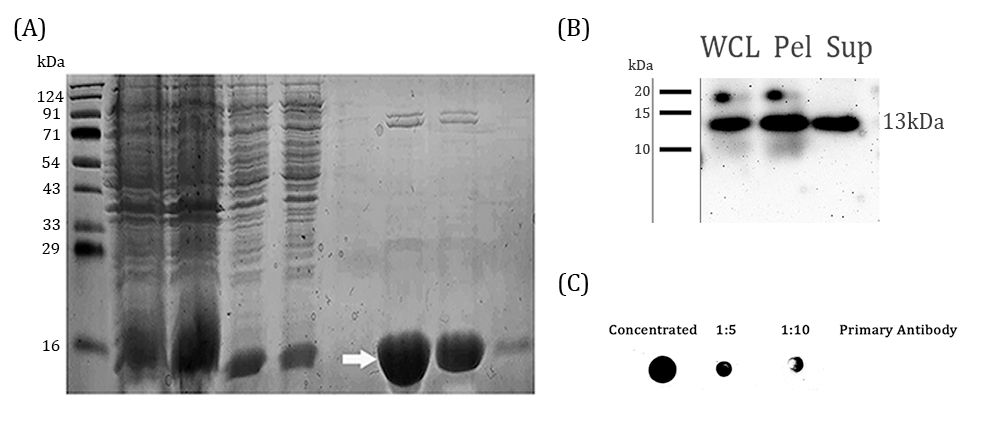

Supplement: Supplementary file 3 [file Image_3.TIF]

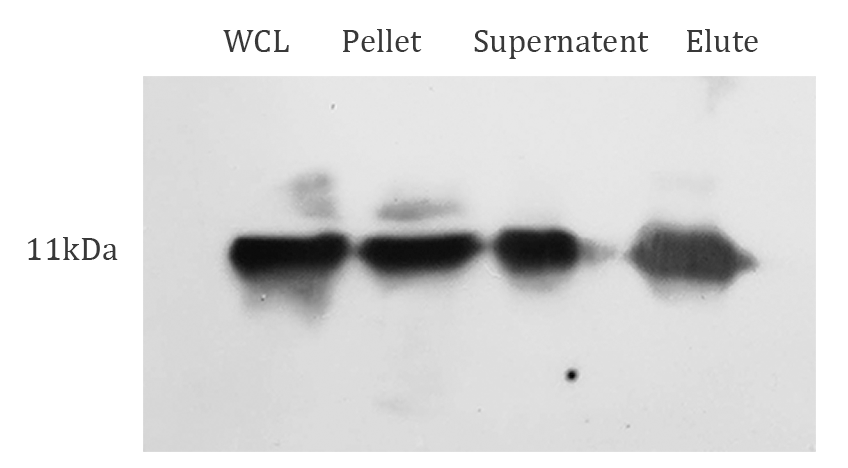

Supplement: Supplementary file 4 [file Image_4.TIF]
