# Supplementary material for: DNA-Binding Properties of YbaB, a Putative Nucleoid-Associated Protein From Caulobacter crescentus
Source: Front Microbiol. 2021 Oct 28;12:733344. doi: 10.3389/fmicb.2021.733344 (PMC8581549; doi:10.3389/fmicb.2021.733344)
Supplement: Supplementary file 5 [file Data_Sheet_1.pdf]

# Supplementary Data

# DNA binding properties of YbaB, a putative nucleoid associated protein from *Caulobacter crescentus.*

**Parul Pal[1], Malvika Modi[1], Shashank Ravichandran[2], Ragothaman M Yennamalli[2] and Richa Priyadarshini[1*]**

[1] Department of Life Sciences, School of Natural Sciences, Shiv Nadar University, Gautam Buddha Nagar, Uttar Pradesh, India

[2] Department of Bioinformatics, School of Chemical and Biotechnology, SASTRA Deemed to be University, Thanjavur, Tamil Nadu, India

ORCID:

Richa Priyadarshini: 0000-0002-8613-9438

Ragothaman M. Yennamalli: 0000-0002-3327-1582

* Corresponding author

**Correspondence and reprints**:

**Dr. Richa Priyadarshini**
Department of Life Sciences, School of Natural Sciences, Shiv Nadar University, Gautam Buddha Nagar, Uttar Pradesh, India.
Ph no: +91-120-3819100 Ext. 220
Email: richa.priyadarshini@snu.edu.in (RP)

## Plasmid Construction

**pPAL1:** *ybab*$_{Cc}$ was amplified using primers Fwd: 5'-CCCCCGGTACCAGGAGGATGAAAGACCTCGGCGGCC-3' and Rev: 5'-CCCCCCTGCAGTTAGAACTTCATCCCGGGCAG-3'. The resulting fragment was ligated with vector pJS14 equally treated with KpnI and PstI.

**pPAL2:** *ybab*$_{Cc}$ was amplified using primers Fwd: 5'-CCCCCGGTACCATGAAAGACCTCGGCGGCC-3' and Rev: 5'-CCCCCGCTAGCTTAGAACTTCATCCCGGGCAG-3'. The resulting fragment was ligated with vector pXCFPN-5, equally treated with KpnI and NheI.

**pPAL3:** *ybab*$_{Cc}$ gene was amplified using primers Fwd: 5'-CCCCCGGATCCATGAAAGACCTCGGCGGCC-3' and Rev: 5'-CCCCCGAGCTCTTAGAACTTCATCCCGGGCAG-3'. The resulting fragment was ligated into vector pET28b, equally treated with SacI and BamHI.

**pPAL4:** *ybab*$_{Cc}$ was amplified using primers Fwd: 5'-CCCCCCATATGATGAAAGACCTCGGCGGCC-3' and Rev: 5'-CCCCCGGTACCTTAGAACTTCATCCCGGGCAG-3'. The resulting fragment was ligated with vector pJS14, equally treated with NdeI and KpnI.

**pPAL5:** truncated *ybab*$_{Cc}$ gene was amplified using primers Fwd: 5'-CCCCCGGATCCATGAAAGACCTCGGCGGCC-3' and Rev: 5'-CCCCCGAGCTCATGACCTCGCCCTCGCCGGG-3'. The resulting fragment was ligated into vector pET28b, equally treated with SacI and BamHI.

## Strain Construction

**RP40:***E. coli* wild type strain MG1655 was transformed with pPAL1 recombinant vector.

**RP41:** BL21 (DE3) pLysS was transformed with pPAL3 recombinant vector.

**RP42:** S17-1 carrying pPAL2 was mated with CB15N

**RP43:** S17-1 carrying pPAL4 was mated with CB15N

**RP44:** BL21 (DE3) pLysS was transformed with pPAL5 recombinant vector.

**RP45:***C. crescentus ftsI*(ts) was transduced with RP42 lysate

**RP46:***E. coli* wild type strain MG1655 was transformed with empty pBAD18 vector.

## Figure legends

**Figure S1.YbaB$_{Cc}$ localization in wildtype CB15N.** RP42 cells (CB15N xylX:: pPAL2) carrying CFP-fused YbaB$_{Cc}$ under the control of P*xyl* promoter were cultured at 30°C in PYE medium till 0.1 OD and then induced with 0.3% xylose. Samples were taken at multiple time points (4h,6h,8h,10h,12h) and imaged on agarose-padded glass slides. The figure shows images from 4h sample. At all time-points, YbaB$_{Cc}$ was found to be dispersed throughout the cell length. The scale bar represents 5μM.

**Figure S2.The Electrostatic surface of YbaB$_{Cc}$ protein** (shown here in surface representation) has a patch of positively charged surface (colored blue) that is most likely to interact with DNA. The sequence range is from Arg51 to Ile71. Image made using APBS and PyMOL.

**Figure S3: Confirmation of purified YbaB$_{Cc}$ protein.** RP41 cells were used to overexpress YbaBcc protein via IPTG induction to carry out purification of the full length YbaB$_{Cc}$ protein. **(A)** Coomassie stained SDS-PAGE gel showing overexpression of YbaB$_{Cc}$ from pet28b plasmid. Lane 1 carries protein ladder. Protein band (13kDa, arrow) is present in sample lanes: Whole cell lysate (lane 2), Pellet fraction (lane 3), Supernatant fraction (lane 4), lanes 5 and 6 are flowthrough and wash buffer samples collected from protein purification process and lanes 7-9 show elution fractions carrying purified protein. **(B)** Western Blot showing 13kDa band corresponding to YbaB$_{Cc}$. **(C)** Dot Blot of purified YbaB$_{Cc}$. 5 µl each of concentrated purified YbaB$_{Cc}$ , 1:5, 1:10 dilutions of the protein and Anti-his antibody (control) were spotted onto a nitrocellulose membrane and probed with anti-his antibody.

**Figure S4: Confirmation of purified truncated YbaB$_{Cc}$ protein.** Western Blot showing 11kDa band from whole cell lysate (WCL), pellet, supernatant and elution fractions.
